# A case of pacing lead induced clinical superior vena cava syndrome: a case report

**DOI:** 10.4076/1757-1626-2-7477

**Published:** 2009-06-23

**Authors:** Mukesh Singh, Sabry K Talab

**Affiliations:** 1 Department of Medicine, Blackpool Victoria HospitalBlackpool, LancashireUK; 2 Department of Internal Medicine, Rosalind Franklin University of Medicine & Sciences, Chicago Medical SchoolNorth Chicago, IllinoisUSA

## Abstract

**Introduction:**

Transvenous pacing is a relatively safe treatment with a low complication rate, but serious thromboembolic complications have been reported to occur in 0.6% to 3.5% of cases. Superior vena cava obstruction syndrome is generally an uncommon but serious complication occurring in <0.1% of patients. However, when it occurs it carries with it significant morbidity and mortality.

**Case presentation:**

A 51-year-old lady with long history of DDD permanent pacemaker presented following a mechanical fall. She had no obvious injuries, and was hemodynamically stable. General examination revealed features suggestive of Superior vena caval obstruction which was later confirmed by imaging. She was treated with long term oral anticoagulation with good clinical improvement.

**Conclusion:**

Superior vena cava obstruction in patients with transvenous pacing leads, although rare, is a well recognized complication. With growing elderly population and increasing number of procedures performed, more and more people with permanent pacemaker are likely to be encountered in clinical practice. One should carefully look for thromboembolic complications during follow-up in patients with transvenous pacemaker leads, as it has implications for future management and carries significant morbidity and mortality.

## Introduction

Although transvenous pacing is a relatively safe treatment with a low complication rate [1], serious thromboembolic complications have been reported to occur in 0.6% to 3.5% of cases [2]. Superior vena cava obstruction [SVCO] syndrome is generally an uncommon but serious complication occurring in <0.1% of patients [3-6]. Fortunately, most patients remain asymptomatic and subclinical because of the development of an adequate venous collateral circulation. Venous obstruction often first becomes apparent during pacemaker lead revision, when difficulty in passing the new pacing lead is encountered. In this report, we present a case of pacemaker associated superior vena caval obstruction syndrome and a brief review of literature.

## Case presentation

This 51-year-old Caucasian lady presented following a mechanical fall. Nine years previously she had had a DDD permanent pacemaker implanted on her left hand side for stokes adams attacks due to second degree atrio-ventricular block. One year ago, she presented with her pacemaker eroding through the skin and then had another pacemaker implantation on right hand side. Old atrial lead was extracted while ventricular lead could not be extracted and was cut short to be left in situ.

This time she presented following a mechanical fall. On examination, her vital signs were stable. General examination revealed a raised jugular venous pressure, distended neck and chest veins and right arm swelling, consistent with SVCO. She had no clubbing, lymphadenopathy, or breast lumps. The rest of her systemic examination was unremarkable.

**Figure 1 (top) & 2 (bottom).**
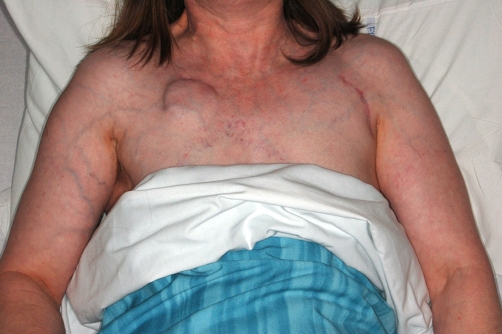

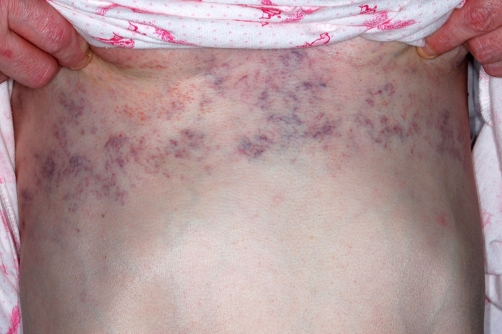
Patient photographs showing pacemaker and dilated veins.

**Figures 3 & 4.**
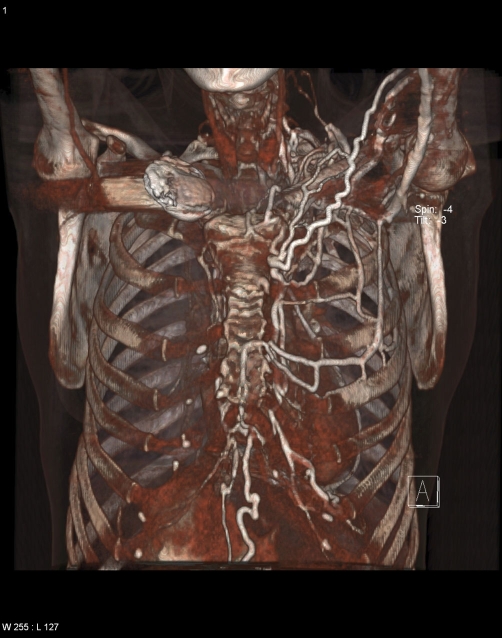

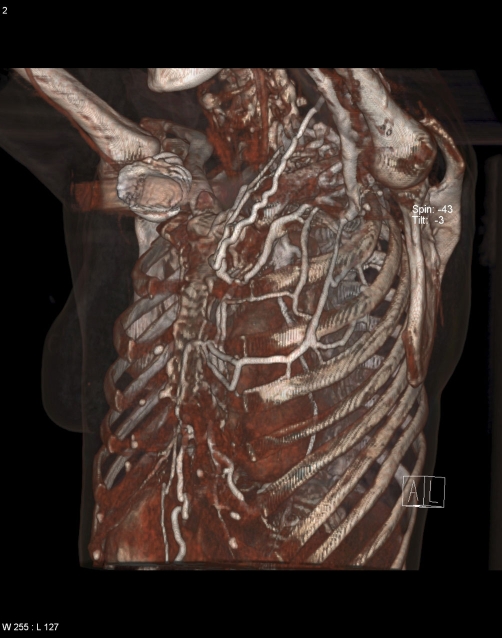
Anterior-posterior **(Figure 3, top)** and oblique **(Figure 4, bottom)** three dimensional volume-rendered images from a contrast enhanced computed tomographic scan demonstrating numerous dilated superficial chest veins over the left chest.

**Figure 5.**
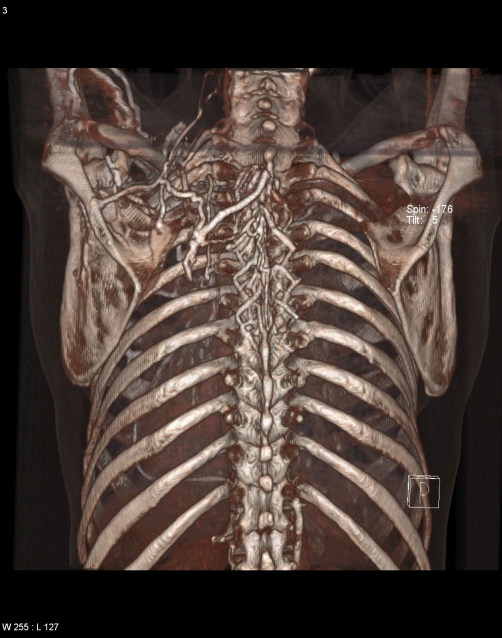
Three dimensional volume-rendered images from posterior aspect showing dilated and tortuous collaterals.

**Figure 6.**
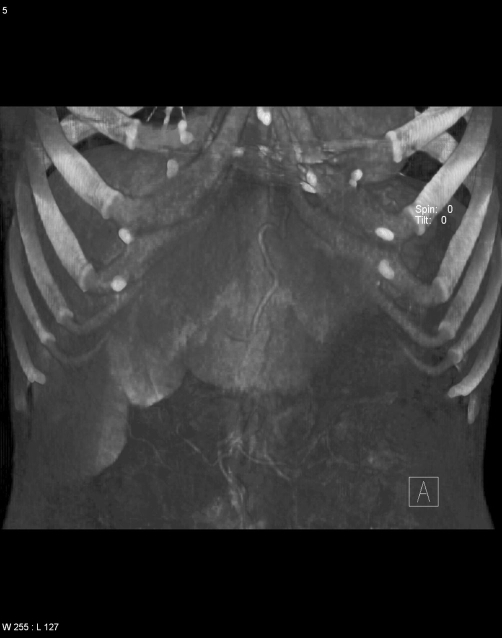
Dilated and tortuous collaterals in the epigastrium.

Her chest X-ray showed the pacemaker leads in situ (Figure 1). Computer tomography scan (Figures 2-6) confirmed the presence SVCO and there was no evidence of underlying malignancy. She was treated with low molecular weight heparin followed by long term oral anticoagulation with warfarin. She was subsequently seen in the cardiology clinic eight weeks later for further evaluation. By this time signs of SVCO have improved and discussions with the cardiothoracic surgeons a decision was made that vascular intervention was not warranted and that she should continue with long term anticoagulation.

**Figure 7.**
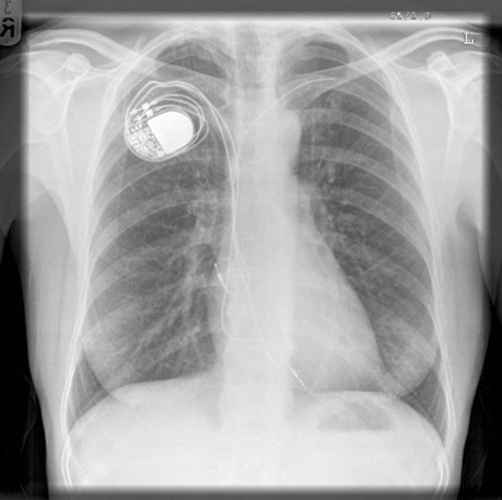
Chest X-ray showing pacemaker and pacing leads.

## Discussion

There are variable reports about the incidence of venous obstruction after pacing lead implantation ranging from 0.6% to 30% [7,8]. Much of this difference can be explained on the basis of differences in definition. Despite relatively high incidence of documented venous obstruction, most patients remain asymptomatic because of the development of an adequate venous collateral circulation [4-6]. In contrast to an incidence of between 8% and 21% of occlusion of the subclavian or brachiocephalic vein in these studies, the incidence of pacemaker induced SVCO syndrome is reported to be very low, at 0.03-0.4% [9,10]. However, when it occurs it carries with it significant morbidity and mortality.

The mechanism is believed to be mechanical stress induced by transvenous leads, eliciting vascular wall inflammation and fibrosis ultimately leading to venous thrombosis and occlusion [11]. Although no clear risk factors have been identified, several predictors of venous occlusion in this setting have been reported as shown in Table 1 [12,13]. On the other hand, long-term anticoagulation therapy seems to offer a protective effect [13].

No significant differences was observed between obstruction and non-obstruction groups in terms of age, sex, cardiothoracic ratio, left atrial dimension, left ventricular ejection fraction, baseline heart diseases for indication of pacemaker implantation, or number and body size of pacing leads [5]. Da Costa et al showed that previous use of a temporary pacemaker and left ventricular ejection fraction of 0.40 or less increased the risk venous thrombosis 6 months after permanent pacemaker insertion [14].

Another recently published study, looking at 100 consecutive patients for elective permanent transvenous pacemaker, did not find any significant difference between two groups in the incidence of venous abnormalities according to the route of entry, the lead insulation or the total number of the implanted leads [6].

Data regarding treatment options for SVCO are limited. Contrary to the treatment of SVCO caused by malignant tumors, treatment for benign causes of superior vena cava obstruction is often protracted, punctuated by multiple episodes of recurrences. These causes include arterio-venous shunt, central lines and cardiac pacing wires. Venous angioplasty and stenting in these patients are often repeated, with cumulative patency approaching 80% at 2 years [15,16].

In cases related to cardiac pacing wires, removal of the device is not only undesirable [in view of the cardiac arrhythmia], but it is also often impossible and may not relieve the symptoms. The leads, which are insulated by silicon, are covered by endothelium and become incorporated into the vascular wall [4]. The pacing wires can also become incorporated into the heart chambers, making removal both difficult and sometimes dangerous. Thrombosis can also occur along these wires leading to stenoses and occlusions of the great veins [17]. Trauma to the vessel wall during insertion, infection and dual chamber systems are thought to be the predisposing factors [9].

Various modalities of treatment that can be considered include long-term anticoagulation as adopted in our case, thrombolytic therapy, surgical intervention, percutaneous transluminal balloon venoplasty and metallic stent insertion. Options depend on the duration, extent, and site of venous occlusion as well as the accompanying symptoms [4]. A recent review by Bracke et al, looking at the evidence base for lead extraction, concluded that there is no evidence to suggest that properly abandoned leads are a risk factor for venous occlusion and they should not be routinely extracted [18].

**Table 1.** Risk Factors for Pacing lead induce venous thrombosis [12,13]

1. History of device upgrade.
2. Use of temporary endocardial pacemaker wires before the implantation of a permanent device.
3. Presence of multiple endocardial leads.
4. Retention of severed leads.
5. Lead infection.
6. Use of dual coil leads.
7. Hormone therapy.
8. History of venous thrombosis.

## Conclusion

SVCO in patients with transvenous pacing leads, although rare, is a well recognized complication. SVCO can interfere with intravenously administered therapy, monitoring of central venous pressure and revision of a pacemaker lead. With growing elderly population and increasing number of procedures performed, more and more people with permanent pacemaker are likely to be encountered in clinical practice. One should carefully look for thromboembolic complications during follow-up in patients with transvenous pacemaker leads, as it has implications for future management and carries significant morbidity and mortality.

## Abbreviation

SVCO: Superior vena cava obstruction
